# The pathogenesis hypothesis and research progress of CLIPPERS: A literature review

**DOI:** 10.1097/MD.0000000000033211

**Published:** 2023-03-17

**Authors:** Long Cao, Meiping Liu, Li Guo, Mingyan Li, Min Xu, Rui Wang

**Affiliations:** a College of Acupuncture and Massage, Shandong University of Traditional Chinese Medicine, Jinan, People’s Republic of China; b First College of Clinical Medicine, Shandong University of Traditional Chinese Medicine, Jinan, Shandong, People’s Republic of China; c Affiliated Hospital of Shandong University of Traditional Chinese Medicine, Jinan, Shandong, People’s Republic of China.

**Keywords:** chronic lymphocytic inflammation with pontine perivascular enhancement responsive to steroids, inflammation, pathogenesis hypothesis, research progress, treatment

## Abstract

Chronic lymphocytic inflammation with pontine perivascular enhancement responsive to steroids (CLIPPERS) is still a rare autoimmune disease in the world. In recent years, there are more and more reports about the clinical manifestations of CLIPPERS, but the specific etiology and pathogenesis are not clear. In this paper, by collating the literature reported in recent years, in the reported effective treatment cases, we found the current hypothesis about the pathogenesis of CLIPPERS. Three pathogenesis hypotheses: organ-specific autoimmunity; virus infection affects autoimmunity; and helper T lymphocyte 17 mediates autoimmunity. Although it is hypothetical, it is expected to further clarify the pathogenesis, evolution characteristics, and treatment of CLIPPERS, so as to provide a reference for further understanding of the disease. In the future, more observations and studies are needed to further verify the feasibility of the hypothesis. This article expands on atypical clinical manifestations and summarizes treatment options. Hope to provide a reference for clinical diagnosis and treatment of CLIPPERS.

## 1. Introduction

Chronic lymphocytic inflammation with pontine perivascular enhancement responsive to steroids (CLIPPERS) is a chronic inflammatory disease occurring around the pons, midbrain, and cerebellar vessels, mainly characterized by lymphocytic infiltration, and effectively treated with steroid hormones. The disease was first reported by Pittock et al,^[1]^ and it is relatively rare in clinics. While brain stem or cerebellar damage presentations are among the initial signs of CLIPPERS, other symptoms gradually develop as the disease progresses.^[2]^ There is no genetic predisposition for CLIPPERS, which typically manifests in people between the ages of 30 and 60,^[3]^ occasionally in children,^[4]^ and more frequently in men than in women (3:1 ratio).^[5]^ Since Pittock’s initial report, cases have gradually been reported from various parts of the world. A comprehensive search of PubMed and Web of Science, Cochrane Library, China National Knowledge Infrastructure, Wan-fang database, Chinese Biomedical Literature Databases, and other databases with the keyword “Chronic lymphocytic inflammation with pontine perivascular enhancement responsive to steroids” and “CLIPPERS” was conducted from inception until January 15, 2022. By reviewing the effective treatment cases and related reports reported in recent years, we summarize the clinical manifestations and diagnostic criteria of the disease, sort out the pathogenesis hypothesis and improve the characteristics of the disease. Through this review, we can broaden the atypical clinical manifestations of CLIPPERS and improve the differential diagnosis from other central nervous system (CNS) diseases as far as possible.

## 2. Hypothesis of pathogenesis

**Figure 1.**
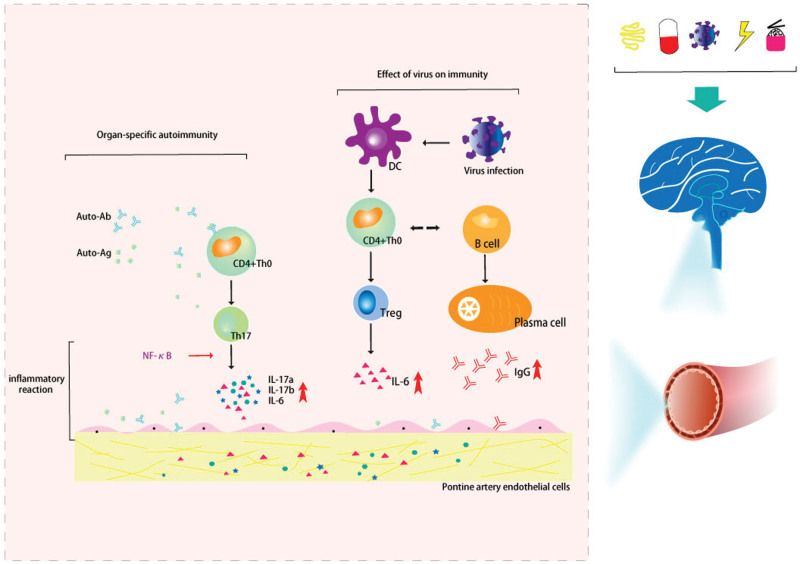
The hypothetical diagram of the mechanism of organ-specific autoimmunity and viral infection affecting Treg and Th17 mediation. Organ-specific autoimmunity: target autoantigens are concentrated in the area around blood vessels and cause perivascular inflammation. Viral infection causes dendritic cells to recognize and accept antigens. Dendritic cells stimulate T and B cells to produce inflammatory mediators such as IL-6, and even excessive production of IgG. In this context, viral infection causes immunogenic inflammation and leads to CLIPPERS. Th17 is formed by the differentiation of THO cells stimulated by IL-23. Th17 has an independent regulatory mechanism of differentiation and development. NF-κB can promote the secretion of IL-17 by Th17. Th17 cells can secrete and produce IL-17a, IL-17f, IL-6, and TNF-α to mobilize, recruit and activate neutrophils. At the same time, IL-17 is mainly an inflammatory cytokine, which can promote the activation of T cells and stimulate epithelial cells, endothelial cells, and fibroblasts to produce a variety of cytokines such as IL-6, IL-8, and other pro-inflammatory factors. It leads to the incidence of CLIPPERS from the above 3 aspects. CLIPPERS = chronic lymphocytic inflammation with pontine perivascular enhancement responsive to steroids. IL = interleukin, NF-κB = nuclear factor-κB, Th17 = helper T lymphocyte 17, TNF = tumor necrosis factor.

Autoimmune disease is a condition brought on by the body’s immunological reaction to a self-antigen, which results in injury to the individual’s own tissues. Three requirements must be met to diagnose an autoimmune disease: the presence of an autoimmune reaction; the exclusion of any chance of a secondary immune reaction; and the exclusion of other causes. Organ-specific autoimmune illnesses and systemic autoimmune diseases are 2 categories of autoimmune conditions. Nowadays, there are only a few case reports that present the CLIPPER’s origin and pathogenesis. Therefore, this paper reviews the literature, sorts out the content, and aggregates the following hypotheses (Fig. 1).

### 2.1. Organ-specific autoimmunity hypothesis

The organ-specific autoimmunity theory was put forth by Pittock et al^[1]^ and Dudesek et al,^[6]^ who believed that target autoantigens are concentrated in the perivascular region and cause perivascular inflammatory responses. Some individuals had higher serum IgE levels, which could indicate that local inflammatory reaction may be connected to the pathogenesis. The CNS venous inflammatory illness may potentially be linked to the involvement of brainstem regions due to the physical distribution of tiny axial meridians in the CNS. A patient with vascular infarction was reported by Tobin et al,^[7]^ who discovered anomalies in both major and small vessels. Immunosuppressive agents and anti-inflammatory drugs have a good effect on it, and premature reduction or withdrawal of the dose will easily cause symptom recurrence. Since specific antibodies and target autoantigens have not been found so far, it is presumed to be an organ autoimmune disease.^[8]^

### 2.2. Hypothesis that viral infection affects autoimmunity

Regulatory T cells (Tregs) are a kind of T cell subset that control the autoimmune response in the body. In the early days, it was also called suppressor T-cells. Tregs as an important factor can maintain immune tolerance, through active regulation to inhibit the activation and proliferation of potentially autoreactive T cells in the normal body, to regulate the immunity of the body. At the same time, abnormal expression of Tregs will lead to autoimmune diseases. The first case of CLIPPERS connected to persistent hepatitis B virus (HBV) infection was reported by Weng et al.^[9]^ Since the prevalence of circulating CD4^+^ CD25^+^ Tregs in peripheral blood was significantly correlated with high serum HBV-DNA viral load, viral clearance function was compromised as Treg numbers rose.^[10]^ Thus, it is proposed that immunological and regulatory T cell (Treg) dysfunction brought on by HBV may contribute to the pathology of CLIPPERS. CLIPPERS were treated with anti-HBV medications, and good effectiveness was attained. Ma et al^[8]^ reported that the serum of a CLIPPERS patient was positive for Epstein–Barr nuclear antigens antibody and Epstein–Barr viral capsid antigen IgG. Interestingly, however, this patient was IgM negative for Epstein–Barr virus (EBV) capsid antigen. Because of Epstein–Barr nuclear antigens with the host proteins (such as neurons protein) with linear and functional homology. Therefore, the body may over-immunize itself against homologous proteins when immunizing against EBV. It has been proposed that one of the reasons for CLIPPERS may be an autoimmune reaction brought on by EBV infection. In a few cases, the condition was said to have developed following an early infection or an influenza vaccination.^[11]^ Xu et al described 1 case of varicella infection and 1 case of influenza A vaccination^[12]^ and also hypothesized that CLIPPERS might be an adverse effect of an inflammatory process following virus infection.

### 2.3. Hypothesis of Th17-mediated autoimmunity

Helper T lymphocyte 17 (Th17) is a newly discovered T subgroup capable of secreting interleukin 17 (IL-17). Th17 is formed by the differentiation of TH0 cells stimulated by IL-23. Th17 has an independent mechanism of differentiation and developmental regulation. IL-17 produced by Th17 cells can effectively mediate the exciting process of neutrophil mobilization, thus effectively mediating tissue inflammation. After binding to the receptor, IL-17 can exert its biological effects through the MAP kinase pathway and the nuclear factor- κB (NF-κB) pathway. Th17 cells can secrete and produce IL-17a, IL-17f, IL-6, and tumor necrosis factor α, which collectively mobilize, recruit and activate neutrophils. Meanwhile, IL-17 is primarily an inflammatory cytokine. IL-17 can promote the activation of T cells and stimulate epithelial cells, endothelial cells, and fibroblasts to produce various cytokines such as IL-6, IL-8, granulocyte-macrophage stimulating factor, and chemical fuel and cellular adhesion molecule 1, this leads to inflammation. Mele et al^[13]^ found that CLIPPERS syndrome is an autoimmune disease mediated by Th17, and found that NF-κB can promote the differentiation and function of Th17, while rifampicin can inhibit the NF-κB pathway and thus inhibit the function of Th17. It was also found that in the absence of corticosteroids, only 3 anti-tuberculosis drugs (rifampicin, isoniazid, and pyrazinamide) could significantly improve the clinical symptoms and imaging changes in patients with CLIPPERS. It is confirmed that the use of these 3 drugs is also effective in the treatment of recurrent symptoms of CLIPPERS. The patients were positive for QuantiFERON-TB, but the positive results were only found in memory T lymphocytes (for tuberculosis antigen) and were not necessarily active tuberculosis. Tan et al^[14]^ used hydroxychloroquine to effectively relieve the clinical symptoms and imaging manifestations of CLIPPERS without recurrence. Rifampicin can inhibit the differentiation and function of NF-κB by inhibiting the enhancement of the nuclear factor kappa-light chain in the activated B cell (Th17) pathway. Hydroxychloroquine affects the differentiation of TH17 and the secretion of IL-17 by regulating micro-RNA590. It is suggested that CLIPPERS may be an autoimmune disease mediated by Th17. Tan also proposed that the remission and aggravation of the patient’s condition are accompanied by changes in the titer of antinuclear antibodies, which may become a monitoring indicator of treatment.

## 3. Pathology

When CLIPPERS patients were examined in vivo in the cerebellum, basal ganglia, thalamus, and pons, the results showed that there was obvious patchy inflammatory cell infiltration of CD3^+^ T and CD4^+^ lymphocytes around the white matter, arterioles, and venules, but a small amount of CD20^+^ B lymphocytes. In general, there is no local demyelination and vascular wall split imaging. At the same time, it has also been suggested that perivascular vasculitis with non-granulomatous vasculitis exists.^[15]^ No characteristic or similar pathological features of neoplastic diseases, histiocytosis, multiple sclerosis, lymphoma, lymphomatoid granulomatosis, viral infection, mycobacteria, Whipple disease, and other diseases were found. Brain biopsy is necessary when the clinical manifestation or imaging is atypical or when the patient is resistant to glucocorticoids.^[6]^

## 4. Diagnostic criteria and clinical manifestations

In 2010, Pittock et al^[1]^ first reported and summarized the clinical manifestations of 8 CLIPPERS patients: subacute gait ataxia, retest, and dysarthria were the main manifestations, and 5 of them reported sensory changes or tingling sensation in the face or scalp. Other symptoms include nonspecific dizziness, nausea, taste disorders, pseudobulbar palsy, tinnitus, tremor, nystagmus, paresis of the lower extremities, loss of sensation, and cramps. By studying the traits of 35 suspected CLIPPERS patients, Tobin^[7]^ improved the diagnostic standards for CLIPPERS in 2017. Clinical symptoms: subacute pontocerebellar impairment; sensibility to hormone therapy; and a lack of other diseases that would provide a more appropriate explanation for the clinical symptoms. Magnetic resonance imaging (MRI) findings: on the enhanced scan, several homogenous enhanced nodules with lesions smaller than 3 mm dispersed across the pontine cerebellum were seen on the MRI, but no ring enhancement or mass effect was present. After hormone therapy, the enhanced nodules dramatically improved, and the aberrant T2 sequence signals could not significantly outperform the increased sequence signals. If both the clinical and MRI criteria are satisfied, possible CLIPPERS can be confirmed. To help with the diagnosis of ruling out other illnesses, Taieb et al^[5]^ outlined warning indicators that do not support CLIPPERS in 2018. They also presented 3 hypotheses for CLIPPERS: CLIPPERS only is a clinical preexisting or abnormal manifestation of definite diseases, such as autoimmune gliosis, primary angiitis of central nervous system, systemic and localized CNS lymphoma, and possibly other diseases; CLIPPERS may be a pre-lymphoma state that may progress to high-grade lymphoma with similar or similar pathophysiological processes as described in lymphomatous granulomatosis; and CLIPPERS may correspond to an unknown inflammatory disease. Bi et al^[16]^ indicated in 2019 that it is significant for the clinical diagnosis of CLIPPERS to meet the following criteria: subacute or progressive signs of cerebellar and brainstem damage (such as dyslexia, ataxia, diplopia, abnormal facial sensation, etc); there are patchy abnormal signal shadows in specific regions (brainstem, cerebellum, and around the fourth ventricle, etc) and enhanced the typical “peppering-like” enhancement foci (diffuse spots, irregular lines) on MRI. According to some studies, the spinal cord, basal ganglia, and other areas may be affected; it responds very well to steroid medication; brain biopsy revealed clear signs of inflammatory T lymphocyte infiltration around blood vessels; and there are other clinically known lesions ruled out.

### 4.1. Typical symptoms and signs

Gait ataxia, dysarthria, diplopia, and sensory abnormalities are the main clinical manifestations of CLIPPERS. These symptoms can be a combination of distinct symptoms in the above areas, although they seldom appear as a single symptom (such as abnormal gait, dysphagia, eye movement disorder, facial numbness, etc). Ataxia and diplopia as the first symptoms of most CLIPPERS, most patients have varying degrees of ataxia and diplopia, unilateral and bilateral will occur. The positive results of the rotation test, finger-nose test, and closed eyes indicate cerebellar damage. Babinski positive indicates that the function of the pyramidal tract is impaired, decreased muscle tone and dull tendon reflex are more common in lower motor neuron lesions. Cerebellar ataxia is often accompanied by nystagmus, dystonia, and dysarthria, which is seen in cerebellar vascular lesions. Lesions in the brain stem and cerebellum can also lead to diplopia. Bilateral cortical medullary tract damage leads to the pharynx and larynx muscle and vocal cord paralysis (pseudobulbar paralysis) lesions in cerebellar vermis or brainstem pathways connected with the cerebellum lead to ataxia dysarthria. Delayed dysarthria can also be caused by spinal neuropathy of the brain nucleus and/or brain nerve and respiratory muscle, which dominates the muscles of the pronunciation and articulation organs. When the lesion affected the cortical brainstem tract, the patient developed dysarthria. Lateral facial numbness is common in the ipsilateral upper spinal trigeminal nucleus or contralateral spinal thalamic tract and thalamic cortical tract. When the lesion on one side of the subroutine part of the lateral medulla oblongata damaged the spinal nucleus of the trigeminal nerve and the spinothalamic tract from the contralateral side, the ipsilateral facial and contralateral limb sensory disturbance, that is, crossed sensory disturbance.

### 4.2. Nonspecific symptoms and signs

Vertigo, nausea, tinnitus, hearing loss, cognitive dysfunction, taste problems, pseudobulbar palsy, action tremor, spastic paresis, dysphagia, and the frontal lobe release sign are additional nonspecific symptoms. At present, the initial symptoms of CLIPPERS are relatively fixed. In addition to the above symptoms, Tao et al^[17]^ reported 1 case of horizontal eye movement disorder as the initial manifestation. Some studies have reported epilepsy as the initial symptom, cortical involvement in addition to pons and cerebellum symptoms,^[18]^ and even CLIPPERS syndrome with trigeminal neuralgia as the initial symptom.^[19–21]^ Syed et al^[22]^ found that the inflammatory proliferation of CD4^+^ T cells involved the skin and caused multiple lung and skin lesions. Wang et al^[23]^ reported a case with stroke-like symptoms as the initial manifestation. The symptoms occurred during the development of the disease, such as headache, diplopia, nystagmus, cerebellar ataxia, hemiplegia, and paraesthesia. Zhang and colleagues identified a CLIPPERS patient with the main clinical features of the regional postpartum syndrome,^[24]^ which is considered a novel clinical characteristic of the disease, distinguishing it from illness on the spectrum of neuromyelitis optica. Brain biopsy is an invasive test that is only studied if it cannot be identified from other diseases, whereas in clinical identification, it is usually required for a careful diagnosis that a large number of noninvasive tests.^[12,25]^

## 5. Imaging performance

The lesion showed long T_2_ and fluid-attenuated inversion recovery sequence speckle-like high signal foci on MRI plain scan and nodular or “peppering-like” speckle-like enhancement on the enhanced scan. The typical punctate-enhancing lesion^[1,5,7,16]^ is following: its size is <3 mm; its shape is homogeneous rather than annula; it is located in the pons, cerebellum, and spinal cord; without mass effect; its response to steroids; and the presence of small foci of T2 hyperintensity in the corresponding area. Pittock et al^[1]^ first proposed that CLIPPERS imaging mainly involves the pons, cerebellum, spinal cord, basal ganglia, and cerebral white matter. As more and more cases have been reported, we find that the disease involves more than the above parts. The number and size of the lesions were proportional to the distance from the pons. In some patients, the internal capsule, thalamus, corpus callosum, and even cortex were involved.^[1,18]^ MRI is not only used as a reference for diagnosing CLIPPERS but also as the preferred method for evaluating disease progression and treatment effects.^[16]^ By searching the literature, most of the lesions in CLIPPERS patients had no distinct mass effect. Only 1 case showed obvious lumps of the cerebellum and herniation of the cerebellar tonsils in the head of the CLIPPERS patient.^[26]^ The distinct enhancement of the lesion indicates the destruction of the blood-brain barrier, but angiogenic edema is rare.^[6]^ Despite the obvious enhancement of the lesions, there was no abnormality in the angiography of the brain and neck, especially venography.^[27]^ When the clinical symptoms are not obvious, MRI is the strong evidence for the diagnosis of CLIPPERS.^[28]^ There was no positive significance in chest computed tomography (CT), abdominal and pelvic CT, whole-body positron emission tomography, prostate ultrasound, mammography, chest X-ray, cerebral angiography, and whole-body gallium scan.

## 6. Laboratory investigation

Cerebrospinal fluid (CSF) indicated inflammatory alterations upon laboratory analysis. The level of IL-6 in the CSF will become a novel biomarker of CLIPPERS activity,^[17]^ and CSF and biochemical evaluation revealed modest anomalies. Patients with CLIPPERS did not show any significant changes in their serum levels of tumor markers, anti-neuron antibodies, auxiliary proteins, or angiotensin-converting enzymes. A skin biopsy will serve as the histopathological foundation because the disease will affect the skin.^[22]^ Brain biopsy will be important and necessary if CLIPPERS cannot be specifically ruled out.^[29]^ Huang et al reported a case of a weakly positive Pan test in CSF, suggesting a slight increase in CSF protein.^[3]^ Oligoclonal bands were detected in CSF in about 20 to 37.5% of patients with CLIPPERS.^[1,30]^ Although the CSF and blood of CLIPPERS patients showed mild abnormalities, there was no specific significance. CSF and hematology tests ruled out infection, vasculitis, paraneoplastic syndrome, lymphoma, sarcoidosis, and other autoimmune diseases.

## 7. Differential diagnosis

The MRI findings of some diseases may be similar to those of CLIPPERS, such as multiple sclerosis, lymphomatoid granulomatosis, CNS vasculitis, primary CNS lymphoma, cerebral arteriovenous fistula, neuromyelitis optica, concentric sclerosis, clinically isolated syndrome, paraneoplastic neurological syndrome, and so on. Therefore, the diseases similar to CLIPPERS findings are called mimics. In addition to the clinical and imaging “red flag” represented by cortical nervous system signs and pons lesions with necrosis, high-dose corticosteroids do not improve the first or recurrent symptoms of mimics is also a strong diagnostic identity.^[31]^

### 7.1. Differential diagnosis with CLIPPERS-mimics

Multiple sclerosis is an autoimmune disease characterized by white matter inflammatory demyelinating lesions of the CNS. Recurrence and remission, as well as numerous temporal and spatial symptoms, are distinctive characteristics of multiple sclerosis. MRI shows multiple patulous demyelinating lesions vertically and symmetrically distributed in bilateral lateral ventricles. Lymphomatoid granulomatosis is a rare type of vasculitis and granuloma reaction. The disease is characterized by vascular nutrition, vascular destructive lesions, and granuloma reaction in various tissues, accompanied by extensive atypical lymphoproliferative infiltration. It is more frequent in elderly adults. The disease is often characterized by multiple system involvement, poor prognosis, and short survival, mainly affecting the lungs, but also affecting other tissues (mostly lung nodules and skin erythema). The enhanced MRI scan may also exhibit “peppering and salt” enhancement when the brain stem and spinal cord are implicated. Mainly the meninges and brain parenchyma’s middle or small blood arteries are affected by primary CNS vasculitis. The typical manifestation of primary CNS lymphoma includes headache, cognitive decline, local kitchen meridian function defect, and characteristic “Jahren-like” changes in cerebral angiography.^[32]^ CNS lymphoma is common in immunodeficient patients. CNS lymphoma has multiple lesions with unclear boundaries and is often distributed along the midline of the brain parenchyma. The magnetic resonance spectroscopy choline/n-acetyl aspartate ratio is greatly enhanced, the MRI enhancement is uniform, there are “satellite foci” and a “slit sign,” and the sensitivity to hormones is low. The main manifestations of neuromyelitis optica are the simultaneous or successive occurrence of optic neuritis, acute transverse or disseminated myelitis, and the characteristic MRI findings are long segmental inflammatory demyelinating lesions of the spinal cord. Concentric sclerosis mainly showed onion-like or tree-ring-like changes in the white matter of the frontal, parietal, occipital, and temporal lobes. The characteristics of the clinically isolated syndrome are isolated optic neuritis, brainstem encephalitis, myelitis, and so on. The lesions are characterized by small disseminated lesions in time or larger lesions formed by the fusion of 1 or more foci. Brainstem encephalitis is often confused with CLIPPERS, but no enhancement or no obvious enhancement on enhanced MRI is the main distinguishing point. Paraneoplastic neurological syndrome is the distant effect of cancer on the nervous system, rather than a group of syndromes in which cancer directly invades and metastasizes to nerve and muscle tissues. The paraneoplastic neurological syndrome is more common in digestive tract tumors, breast cancer, small cell lung cancer, gynecological tumors, and other malignant tumors. They often have similar clinical manifestations to CLIPPERS: walking, dysphagia, muscle weakness, motor incoordination, slurred speech, memory loss, vision problems, sleep disorders, dementia, seizures, loss of sensation in upper and lower limbs, and dizziness, which can be identified by enhanced MRI and tumor markers.^[33]^

### 7.2. Differential diagnosis from other CNS diseases

Degenerative diseases, such as multiple system atrophy, have a chronic onset and gradual progress, with clinical manifestations of ataxia, but its characteristic MRI is olivary pontine cerebellar atrophy.^[34]^ Tumor diseases: pathological examination can see perivascular lymphohistiocytic infiltration, of which imaging can show mass effect and ring enhancement, and the effect of hormone therapy is not good or improved for the first time, and the disease progression worsens after drug withdrawal.^[4]^ Preliminary differential diagnosis can be made by tumor marker examination. Most infectious diseases have an acute onset, with clinical manifestations similar to those of CLIPPERS, such as fever, dizziness, nausea, ataxia, and even disturbance of consciousness. CSF mostly has biochemical changes, and imaging rarely shows spots centered on the pons.^[35]^ Pontine infarction is shown because the artery that supplies pontine produces sclerosis and forms a thrombus and causes the blood supply to be insufficient or a blood circulatory obstacle. The main symptoms are eye movement disorder, ataxia, contralateral deviant body sensory disorder, limb paralysis, lethargy or lethargy, and cerebral rigidity. The clinical manifestations of CLIPPERS are similar to those of CLIPPERS, and enhancement and onset time are the main differentiators. Neurosarcoidosis may also have subacute onset, but the lesions are more than the subthalamus, or the whole brain hemisphere, cerebellum, and brainstem. Nodular changes are common in lung CT, nodular or ring enhancement can be seen in enhanced imaging, and edema can be found around the lesions.^[36]^ Positive test results can be used for differential diagnosis even though systemic immunological disorders affecting the CNS may exhibit comparable symptoms, the majority of which are accompanied by damage to other organs. According to Taieb et al research,^[5]^ when the noninvasive examination is still questionable, long-term follow-up and pathological results are still required to confirm. Autoimmune glial fibrillary acidic protein (GFAP) astrocytopathy was initially proposed by Lennon et al in 2016. Before 2016, CLIPPERS reported if they did not undergo immunofluorescence detection, they might have autoimmune GFAP astrocytic illness. Due to the similar clinical and imaging manifestations of the 2 diseases, the detection of GFAPα-antibody at the early stage and every new attack will be the breakthrough point to distinguish these 2 rare diseases.^[37]^

## 8. Treatment

There are currently no CLIPPERS treatment guidelines. Clinical use of steroid-based immunosuppressive therapy, intravenous infusion of high-dose dexamethasone, methylprednisolone, or oral high-dose prednisone pulse therapy.^[1,6]^ After high-dose hormone pulse therapy, the clinical symptoms and imaging manifestations have been improved to varying degrees. After a large dose of hormone pulse therapy, the clinical symptoms and imaging findings have different degrees of improvement. Without long-term maintenance therapy with immunosuppressive agents or low-dose hormones, clinical symptoms will recur rapidly.^[31]^

### 8.1. Early symptoms (within a week of diagnosis)

Studies have shown that in the early stage of CLIPPERS treatment, compared with high-dose intravenous corticosteroids, the use of prednisone (1 mg/kg/d) or 6 courses of cyclophosphamide and azathioprine or immunoglobulin is more effective in improving clinical symptoms. And early use of hormone therapy is necessary.^[1,38,39]^ The early use of high-dose hormone pulse therapy was almost uncontroversial. Through literature review, it was found that timely intravenous injection of methylprednisolone (1 g daily for 5 days) at the onset of the disease could significantly improve gait ataxia, retest, paresthesia, and dysrhythmia, and the number and size of enhanced foci in imaging were alleviated.^[1]^ There were also pathological reports that the symptoms of horizontal eye movement inability, dizziness, and gait instability completely disappeared after the steps of methylprednisone (500 mg/7 d, 250 mg/7 d, 120 mg/7 d).^[40]^ Taieb et al^[41]^ proposed that short-term high-dose hormone shock therapy (500 mg or 1 g, 3 or 5 days) should be started as soon as possible in the acute phase. Intravenous methylprednisolone injection could be extended for 10 days if the clinical effect was not distinct.

### 8.2. Symptom remission period (after hormone pulse therapy)

After high-dose hormone shock therapy, the clinical symptoms and imaging changes are significantly improved, and oral corticosteroid treatment is usually used to prevent a recurrence. However, the side effects of long-term corticosteroid use (such as hypertension, hyperlipidemia, diabetes, osteoporosis, etc) should not be ignored. Therefore, Taieb et al^[31]^ proposed a relatively scientific stepwise dose reduction scheme: at the beginning, oral prednisone was given at a dose of 1 mg/kg/d for 4 to 8 weeks, then reduced by 10 mg to 20 mg/d every 2 weeks, then reduced by 2.5 mg to 10 mg every 4 weeks, and finally reduced by 1 mg to 5 mg/d every 4 weeks. Physical indicators should be observed during continuous steroid use, and brain MRI should be performed every 3 months, as imaging progress often precedes clinical findings. Taieb proposed a stepwise reduction to let steroid hormone eventually reduce to 5 mg/d. However, recent studies have shown that oral corticosteroids >20 mg or even >30 mg ensure a low recurrence rate.

How to treat CLIPPERS patients who are unable to use and tolerate steroid therapy will be the focus of future research. Dudesek et al^[6]^ believe that oral glucocorticoids and glucocorticoid retention immunosuppressants are necessary to maintain long-term treatment for the remission of CLIPPERS. Clinically, immunosuppressants can be added to reduce adverse reactions, delay the progression of the disease and prevent recurrence.^[42]^ Although there are no drugs recommended for the treatment of CLIPPERS syndrome except glucocorticoids, there are cases of medium- and long-term use of other drugs for alternative therapy. One patient took rituximab monoclonal antibody for half a year, which could significantly improve the disease with good stability and no side effects.^[43]^ One case reported that although the clinical symptoms and imaging findings had disappeared completely before receiving weekly oral leflunomide (100 mg), there was no recurrence of symptoms during the follow-up of the following year.^[44]^ Leflunomide is an active metabolite of drugs for the treatment of rheumatoid arthritis, inhibits dihydroorotate dehydrogenase, affects pyrimidine metabolism, and regulates T cells. T cells play an important role in the pathogenesis of CLIPPERS. Using leflunomide to control CLIPPERS has a good relative prognosis.^[40]^ IFNβ1a as a glucocorticoid-sparing therapy, can significantly reduce the recurrence rate, reduce nervous system sequelae, and can change glucocorticoids to low-dose oral (30 mg/2 d).^[6,45]^ Our team reported a case of CLIPPERS treated by acupuncture combined with hormone without recurrence.^[46]^ Acupuncture not only suppresses inflammatory response by reducing the production of NLRP3mRNA through Nrf2/HO-1 signaling pathway but also reduces the inflammatory response.^[47]^ It can also help prevent spinal cord injury.^[48]^ Because of the viral infection hypothesis and the close relationship between nervous system diseases and high viral load, Weng et al^[9]^ suggested that anti-HBV drugs should be added to treat CLIPPERS and closely monitor the activity of the HBV. Hou et al^[40]^ reported a case of CLIPPERS whose symptoms were successfully relieved by hydroxychloroquine for 4 years. The symptoms of this case were improved 2 months after anti-tuberculosis treatment and completely relieved 18 months after anti-tuberculosis treatment. The symptoms of this case were improved 2 months after anti-tuberculosis treatment and completely relieved 18 months after anti-tuberculosis treatment. Tan et al^[14]^ reported that hydroxychloroquine treatment of CLIPPERS can not only quickly relieve the disease, but also effectively prevent recurrence with fewer adverse reactions. The results of Mele et al^[13]^ show that the symptoms of patients are relieved after receiving anti-tuberculosis drugs such as rifampicin and isoniazid, so it is considered that anti-tuberculosis therapy may have a certain effect on CLIPPERS syndrome, but it remains to be confirmed by further trials. However, there are no randomized controlled trials to verify the safety and effectiveness of the above treatment cases.

## 9. Prognosis

Most of the patients with CLIPPERS had a good prognosis after continuous low-dose corticosteroid therapy, and some patients relapsed after stopping corticosteroid or low-dose corticosteroid therapy. The treatment results of most recurrent patients also proved that re-hormone therapy was still effective for patients with relapse.^[1,34]^ The therapeutic effect can be monitored by regular clinical examination and continuous head MRI (once every 3 months). It has also been reported that CLIPPERS was diagnosed early but developed into other diseases at a later stage. Jin et al^[49]^ reported a case of early diagnosis of CLIPPERS but developed a case of lymphomatoid granulomatosis 6 months later. Lei et al^[50]^ reported a patient with CLIPPERS whose symptoms disappeared after treatment and was diagnosed with anti-myelin oligodendrocyte glycoprotein-IgG associated disorders after repeated recurrence and proposed that anti-myelin oligodendrocyte glycoprotein-IgG associated disorders is a form similar to CLIPPERS. Taieb et al^[41]^ conducted a comparative study on whether patients with CLIPPERS syndrome were treated with immunosuppressants for a long time. Some patients were given an intravenous infusion of high-dose glucocorticoid without maintenance therapy, while others were given long-term immunosuppressive maintenance therapy after an intravenous infusion of high-dose glucocorticoid. The study found that the slower the hormone reduction during hormone maintenance therapy, the longer the relapse time. And in the later stage of treatment, when the daily dosage of glucocorticoid was >20 mg, the symptoms of the patients were stable. And the patients who insisted on glucocorticoid maintenance therapy for a long time in the later stage had fewer clinical symptoms and longer intervals than those without maintenance therapy.

## 10. Discussion

More and more cases of CLIPPERS have been reported in recent years. We can consider whether the etiology of the disease is related to race, climate, living environment, diet, customs, and other habits and genetic factors.

The diagnosis of CLIPPERS should be based on characteristic imaging findings, specific clinical manifestations of specific sites, conditional brain biopsies, pathological examinations, a large number of laboratory and CSF examinations, and the exclusion of other diseases. Because of the great possibility of the same disease with a different shadow and different diseases with the same shadow, it is necessary to analyze and differentiate with various examinations. After excluding other laboratory examinations and systemic multi-system diseases, it is not difficult to diagnose and differentiate CLIPPERS, but the real diagnosis depends on pathological means. At present, although the typical clinical manifestations of CLIPPERS have been improved, the nonspecific manifestations are still expanding. In the future, a more detailed and clear diagnosis is needed to improve the diagnostic criteria and clinical manifestations of CLIPPERS and to provide a more accurate basis for clinical diagnosis and treatment. Based on the review of the literature, we put forward the clinical treatment plan for CLIPPERS: timely intervention, early treatment, high-dose hormone pulse therapy (1 g/3 d) in the early stage of the disease, and stable treatment with low-dose prednisone (>30 mg) after symptom relief. If there are no clinical symptoms or aggravation of imaging manifestations after 3 months, long-term maintenance therapy with other immunosuppressants or other drugs can be considered to prevent a recurrence, consolidate the curative effect and improve the quality of life.

At present, the reports on CLIPPERS are mainly case-by-case or retrospective analyses, and the literature on the pathogenesis is rare. We summarize the diagnostic criteria, clinical manifestations, imaging findings, and treatment of CLIPPERS in recent years. This review will help to increase the understanding of the pathogenesis of CLIPPERS, improve the accuracy of clinical diagnosis, and enhance the differential diagnosis of similar diseases. Our research aims: to improve the understanding of CLIPPERS and to reduce the incidence of misdiagnosis and missed diagnosis; to explain the pathogenesis of CLIPPERS from organ-specific autoimmunity, the viral infection affects autoimmunity, and Th17-mediated autoimmunity, which can better guide the clinical use of drugs and further mechanism research. The limitation of the study is that the rarity of CLIPPERS disease increases the difficulty of disease diagnosis, resulting in rare reports about the pathogenesis of the disease. The pathogenesis of CLIPPERS needs to be further studied. This paper collates the typical and atypical clinical manifestations and expands on the atypical clinical manifestations. We try our best to improve the differential diagnosis and hope to provide help for clinical diagnosis and treatment of CLIPPERS.

## Author contributions

**Formal analysis:** Meiping Liu.

**Methodology:** Li Guo, Mingyan Li, Min Xu.

**Writing – original draft:** Long Cao.

**Writing – review & editing:** Long Cao, Rui Wang.
